# Diagnostic Value of Magnetic Resonance Imaging in Fibrodysplasia Ossificans Progressiva

**DOI:** 10.1002/jbm4.10363

**Published:** 2020-04-28

**Authors:** Esmée Botman, Bernd P Teunissen, Pieter Raijmakers, Pim de Graaf, Maqsood Yaqub, Sanne Treurniet, Ton Schoenmaker, Nathalie Bravenboer, Dimitra Micha, Gerard Pals, Arend Bökenkamp, J Coen Netelenbos, Adriaan A Lammertsma, Elisabeth MW Eekhoff

**Affiliations:** ^1^ Department of Internal Medicine section Endocrinology, Amsterdam Bone Center Amsterdam UMC, Vrije Universiteit Amsterdam, Amsterdam Movement Sciences Amsterdam The Netherlands; ^2^ Department of Radiology & Nuclear Medicine Amsterdam UMC, Vrije Universiteit Amsterdam Amsterdam The Netherlands; ^3^ Department of Periodontology Academic Centre for Dentistry Amsterdam (ACTA), University of Amsterdam and Vrije Universiteit Amsterdam The Netherlands; ^4^ Department of Clinical Chemistry, Amsterdam Bone Center, Amsterdam Movement Sciences Amsterdam UMC, Vrije Universiteit Amsterdam Amsterdam The Netherlands; ^5^ Department of Clinical Genetics, Amsterdam Bone Center Amsterdam Movement Sciences, Amsterdam UMC, Vrije Universiteit Amsterdam Amsterdam The Netherlands; ^6^ Department of Paediatric Nephrology Amsterdam UMC, Vrije Universiteit Amsterdam Amsterdam The Netherlands

**Keywords:** ANALYSIS/QUANTITATION OF BONECLINICAL TRIALSDISEASES AND DISORDERS OF/RELATED TO BONEFIBRODYSPLASIA OSSIFICANS PROGRESSIVARADIOLOGY

## Abstract

Using [^18^F] Sodium Fuoride (NaF) Positron Emission Tomography (PET) it is not only possible to identify the ossifying potency of a flare‐up, but also to identify an asymptomatic chronic stage of fibrodysplasia ossificans progressiva (FOP). The purpose of this study was to investigate the diagnostic role of a more widely available imaging modality, Magnetic Resonance Imaging (MRI), which is of special interest for studies in pediatric FOP patients. MRI and [^18^F]NaF PET/CT images at time of inclusion and subsequent follow‐up CT scans of 4 patients were analyzed retrospectively. Presence, location, and intensity of edema identified by MRI were compared with activity on [^18^F]NaF PET. Occurrence or progression of heterotopic ossification (HO) was examined on the follow‐up CT images. Thirteen different lesions in various muscle groups were identified: five lesions with only edema, five lesions with both edema and increased [^18^F]NaF uptake, one lesion with only increased [^18^F]NaF uptake, and two lesions with neither edema nor uptake of [^18^F]NaF. Mild edema, found in three lesions, was present at asymptomatic sites, which did not show increased [^18^F] NaF uptake or progression of HO on consecutive CT images. Moderate edema was found in three symptomatic lesions, with increased [^18^F]NaF on PET and progression of HO on CT. Severe edema was identified in four lesions. Interestingly, two of these lesions did not develop HO during follow‐up; one of these two even gave obvious symptoms of a flare‐up. MRI can identify whether symptoms are the result of an acute flare‐up by the presence of moderate to severe edema. The occurrence of severe edema on MRI was not always related to an ossifying lesion. The additional diagnostic value of MRI requires further investigation, but MRI does not seem to fully replace the diagnostic characteristics of [^18^F]NaF PET/CT in FOP. © 2020 The Authors. *JBMR Plus* published by Wiley Periodicals, Inc. on behalf of American Society for Bone and Mineral Research.

## Introduction

Fibrodysplasia ossificans progressiva (FOP) is an extremely rare disease leading to ankyloses caused by excessive heterotopic bone formation in connective tissue.^1^, ^2^, ^3^ This progressive autosomal dominant disorder is characterized by periodic flare‐ups.^2^, ^4^, ^5^ A flare‐up is thought to start with an inflammatory process in muscles, tendons, and ligaments. Flare‐ups often, but not always, result in formation of heterotopic ossification (HO).^5^ Nowadays, a flare‐up is defined by its symptoms, the most prominent ones being swelling and pain.^5^ Because of the lack of (blood) markers, a flare‐up is still a clinical diagnosis. [^18^F] Sodium fluorde (NaF) Positron Emision Tomography (PET) / Computed Tomography (CT), however, it has become possible to identify which of these flare‐ups will lead to HO.^6^, ^7^ Recently, it was shown that [^18^F]NaF PET/CT can also identify asymptomatic chronic progression of existing HO based on relatively lower, but still increased [^18^F]NaF uptake.^8^ Using this new marker of active and chronic FOP disease, it has become possible to investigate the diagnostic role of other, more widely available imaging modalities such as Magnetic Resonance Imaging (MRI). The absence of ionizing radiation makes this technique especially interesting for the pediatric FOP population.

MRI provides high soft tissue contrast and is able to detect edema, a potential marker of inflammatory stages in FOP.^9^, ^10^, ^11^, ^12^ MRI is currently used in FOP to evaluate the presence of edema early in flare‐ups.^10^ Whether the two stages of FOP, active flare‐ups and asymptomatic chronic FOP disease, have similar inflammatory patterns and corresponding MRI signals is not yet known.

A few FOP case reports have described hyperintense foci on T2‐weighted images during flare‐ups,^9^, ^11^, ^13^, ^14^, ^15^ but whether these hyperintense foci on MRI led to formation of HO was not investigated. Nevertheless, in R206H Acvr1 knock‐in mice, it was shown that sites of HO formation coincided with hyperintense T2 signals. Once HO was formed, the signal subsided. In this animal model, the MRI T2‐signal was in accordance with the [^18^F]NaF PET signal.^16^ For human FOP patients, however, it is not known whether edema on MRI can distinguish between ossifying and nonossifying flare‐ups, as well as between active and asymptomatic chronic FOP disease, as in the case for [^18^F]NaF PET/CT.^8^ Therefore, the aim of this study was to assess whether MRI can identify different stages of FOP. Although ossifying and nonossifying flare‐ups are clinically indistinguishable,^5^ it is possible that the severity of edema on MRI may predict the fate of a flare‐up. In addition, assuming that HO formation is always accompanied by edema, it should be possible to detect the chronic component of disease by MRI.

## Methods

Adult FOP patients, under the care of the FOP expertise center of the Amsterdam UMC, in whom both MRI and [^18^F]NaF PET/CT scans were performed, were included. MRI and [^18^F]NaF PET/CT scans had to be acquired within 14 days of each other to allow for reliable comparisons. In addition, a follow‐up CT scan, obtained at least 6 months after the initial scans, had to be available to assess whether HO had formed. Any additional MRI and [^18^F]NaF PET/CT images, available within the studied timeframe, were included in the analyses as well. Patients were asked to sign a consent form for analyzing and publishing their data anonymously. The study had been approved by the Medical Ethics Review Committee of Amsterdam UMC.

MRI was acquired on a 1.5‐T system (Signa Excite HDxt; GE Healthcare, Milwaukee, WI, USA) and a 3‐T system (Vantage Titan; Canon Medical Systems, Otawara, Japan). MRI scans were obtained either at an annual follow‐up or for a suspected flare‐up. Although different imaging protocols were used, T2 STIR (short‐TI inversion recovery) acquisitions were available for all MRI scans included. MRI parameters are presented in Table 1 of the supplemental material. The intensity of edema was graded on a semiquantitative scale: absent, mild, moderate, or severe.^17^, ^18^, ^19^ Two reviewers (BPT and EB) independently graded the degree of edema. BPT was not involved in clinical care of the FOP patients at the time of image acquisition. Both reviewers were blinded to clinical data. To limit bias, the review of [^18^F]NaF PET and CT data was performed at least 2 months apart from the review of the MRIs. The reviewers were instructed to grade edema in accordance to Davis et al. a juvenile dermatomyositis scoring system for MRI.^17^ Discrepancies were resolved by consensus.

**Table 1:** Demographic Characteristics of the Included Patients

	Sex	Age^a^	Flare‐up^b^	Medication^c^
1	♀	19	m. psoas, m. iliopsoas	Prednisolone, ibuprofen
2	♀	23	**‐**	‐
3	♀	23	Flare‐up bilateral jaw	Prednisolone, ibuprofen
4	♂	20	Suspicion flare‐up jaw	Naproxen

a Age at time of the first MRI.

b Flare‐up during the course of the study.

c Medication taken during the course of the study. This may have been temporarily or continuously.

[^18^F]NaF PET/CT scans were obtained using a Gemini TF‐64 PET/CT scanner (Philips Medical Systems, Best, The Netherlands). Patients were scanned from the top of the skull to the toes. The [^18^F]NaF dose was adjusted to weight (eg, 83 MBq [^18^F]NaF for a 70‐ to 79‐kg patient) and a scan time of 2 min per bed position was used. Uptake of [^18^F]NaF was considered increased for chronic lesions when SUV_peak_ exceeded 8.4.^8^ The method used to analyze [^18^F]NaF PET/CT has been previously described.^8^ For flare‐ups, no SUV_peak_ cut‐off was available, but in practice it is assumed to be 2 to 3 times higher than that for a chronic lesion.^8^ Whole‐body CT images were acquired at 120 kV with a tube current varying between 30 and 60 mAs. CT volumes were obtained for each heterotopic lesion identified. Two reviewers (EB and BPT) independently analyzed [^18^F]NaF PET/CT images. Again, discrepancies were resolved by consensus.

Follow‐up low‐dose whole‐body CT scans were also acquired at 120 kV with a tube current varying between 30 and 60 mAs. The volumes of the various lesions were analyzed to assess whether these lesions progressed during the course of this study. The same two reviewers (EB and BPT) independently analyzed CT images. Discrepancies were resolved by consensus.

Clinical data were evaluated separately to assess clinical signs of the patients at the time of MRI, [^18^F]NaF PET/CT, and follow‐up CT scans. A flare‐up was assessed and confirmed by the physician based on the presence of symptoms, eg, swelling, redness, and pain. When HO progressed in the absence of any clinical symptoms at that site in the last 6 months it was considered chronic.

Interobserver correlation was assessed using Cohen's kappa. Spearman's rho was used for correlation between edema grading and SUV_peak_ values. All statistical analyses were performed using IBM SPSS Statistics for Windows, version 24.0 (SPSS, Inc., Chicago, IL, USA).

## Results

Four FOP patients were included: All four MRI and [^18^F]NaF PET/CT images, and at least one follow‐up CT scan, were available. In fact, for the assessment of these four patients, eight MRI scans, six [^18^F]NaF PET/CT scans, and four follow‐up CT scans were available.

Using MRI, 10 different edematous lesions were found. Increased sodium fluoride uptake was found in only five and clinical signs of a flare‐up were present in only six of the lesions identified by MRI (Table 2). Using [^18^F]NaF PET/CT, six lesions showed increased uptake of [^18^F]NaF. In five lesions, edema was present and for five of those six [^18^F]NaF‐positive lesions patients complained of discomfort. Six sites were identified solely by discomfort that was recognized by the patient as a flare‐up. In four of the six symptomatic sites, edema was demonstrated by MRI. Only three of these six symptomatic sites showed increased [^18^F]NaF uptake on PET. Combining the lesions and sites found with MRI, [^18^F]NaF PET/CT, and clinical signs, 13 distinctive sites were identified and analyzed further.

**Table 2:** Identified Muscle (Groups) by Either Complaints, Edema on MRI, or Increased [^18^F]NaF Uptake on PET/CT

Muscle (group)	MRI edema	[^18^F]NaF PET (SUV_peak_)	Clinical signs	Progression HO volume
Jaw dextra	None	4.1	Present	No
Jaw sinistra	None	2.3	Present	No
Paracostal area dextra	None	**9.8**	Absent	Yes
M. psoas sinistra	Mild	5.4	Absent	No
M. glutei dextra	Mild	1.4	Absent	No
M. glutei sinistra	Mild	2.2	Absent	No
Jaw dextra	Moderate	**28.5**	Present	Yes
Jaw sinistra	Moderate	**26.4**	Present	Yes
M. psoas dextra	Moderate	**32.5**	Present	Yes
M. Iliopsoas dextra	Severe	3.9	Present	No
Mm. adductors dextra	Severe	**48.7**	Present	Yes
M. quadriceps dextra	Severe	**41.9**	Present	Yes
M. gluteus maximus	Severe	2.1	Absent	No

In bold: SUV_peak_ that exceed the threshold of 8.4, as found by Botman et al.^8^

HO = Heterotopic ossification; M. = musculus.

Of the 10 edematous lesions identified by MRI, edema was classified as mild in three, moderate in three, and severe in four lesions (Table 2). There was a good correlation in scoring of edema between the two independent reviewers (Cohen's κ = 0.7; *p* < 0.05).

**Figure 1:**
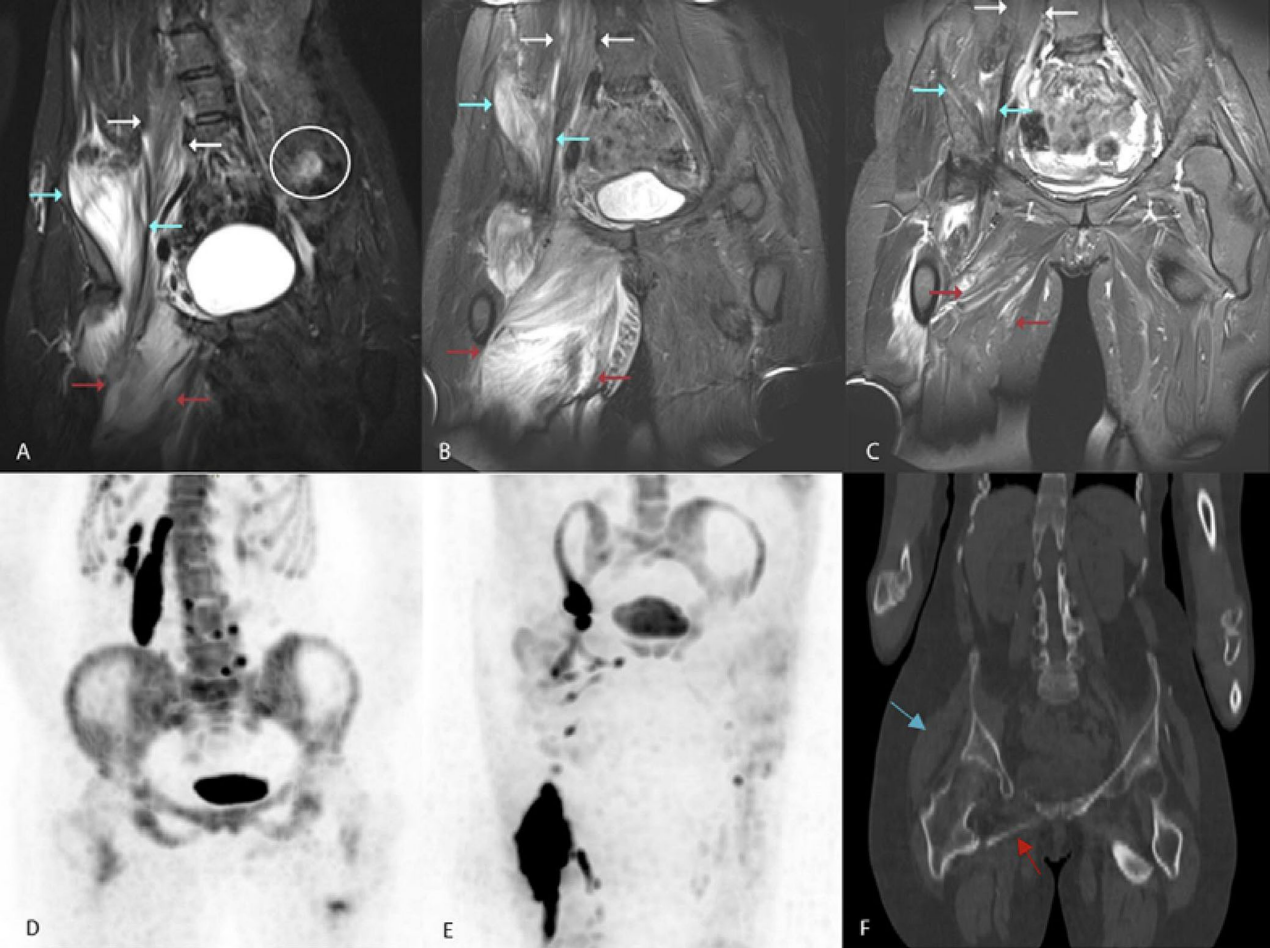
Consecutive MRI scans and [^18^F]NaF PET scans of a patient with several flare‐ups. Coronal MRI T2‐weighted short‐TI inversion recovery (STIR) images are shown of a patient with multiple flare‐ups. Starting in the loin (*A* + *D*), later also the groin (*B*) and upper leg (*C* + *E*). Time in months. *A* and *D*; T = 0. Clinically, a flare‐up in the pelvic area with pain and swelling of the entire right loin. MRI (*A*) showed moderate and severe edema of the musculus psoas dextra (white arrows) and the musculus iliacus dextra (blue arrows), respectively. Also, the musculi adductors (red arrows) showed moderate edema, even though no clinical signs were noted. The MRI showed also an area of nonspecific mild edema (white circle). [^18^F]NaF PET showed increased high uptake of tracer in the psoas muscle, mild uptake in the mm. adductors and no uptake in the iliopsoas muscle. *B*; T = 1. Edema at both the musculus psoas and musculus iliacus diminished, to mild and moderate edema, respectively. Edema intensity at the musculi adductors increased to severe, the patient now reported flare‐up symptoms at the groin too. The mild edema seen in plane A resolved; no calcifications were noted. *C* and *E*; T = 11. Edema at the psoas muscle, and the iliacus and adductor muscles is completely resolved, but new edema formed in the quadriceps muscle (*C*). High [^18^F]NaF uptake in the quadriceps (*E*). *F*; T = 21. Low‐dose whole‐body CT showed heterotopic ossification (HO) in the psoas muscle, HO at the site of the adductor muscles (red arrow), and in the quadriceps muscle. No HO formed in the iliopsoas (blue arrow).

None of the three mildly edematous lesions showed progression of HO. There was no increased uptake of [^18^F]NaF, nor were there any clinical signs reported at these sites. In contrast to these mild lesions, all three lesions with moderate edema were followed by HO progression. In addition, all showed increased [^18^F]NaF uptake and were accompanied by signs of a flare‐up. Interestingly, two of the four sites with severe edema did not develop HO. Although one of these lesions was accompanied with common flare‐up symptoms, such as swelling and pain (Fig. 1A‐C, blue arrows), it did not show increased activity on [^18^F]NaF PET. Interestingly, the adjacent muscle showed moderate edema (Fig. 1A‐1C, white arrows), which did result in HO development. [^18^F]NaF uptake was increased only in the muscle group that showed HO development. Figure 1D‐1F shows the course of the edema over 11 months, with great differences in edema intensity after one month. After 11 months, the edema had completely resolved.

The other lesion with severe edema was a clinically asymptomatic lesion. Again, the [^18^F]NaF PET scan did not show increased uptake of [^18^F]NaF in this area and the successive CT scan did not reveal any HO progression of that particular region.

One site was identified by increased uptake of [^18^F]NaF on PET, but did not reveal any edema. The patient denied having had complaints in that area for at least six months. The follow‐up CT scan, however, revealed progression of HO in that particular lesion. Taking all edematous lesions into account, [^18^F]NaF SUV_peak_ showed no significant correlation with edema intensity (Spearman's ρ = 0.4; *p* = 0.222). In addition, the volume increase of new lesions or with HO progression was weakly related to the intensity of the edema as found by MRI (Spearman's ρ = 0.6; *p* = 0.244).

Apart from lesions identified by MRI and [^18^F]NaF PET, two sites were analyzed because of clinical complaints suggesting a flare‐up. MRI did not show edema at either site, neither did [^18^F]NaF PET show increased uptake of [^18^F]NaF. In addition, there was no HO progression on the subsequent CT scan. The patient's complaints resolved within a couple of weeks without intervention, suggesting a different underlying mechanism. Taking [^18^F]NaF PET/CT as the golden standard, MRI had a sensitivity of 83% and a specificity of 29% to detect FOP activity leading to HO formation. Positive and negative predictive values of MRI in this study were 50% and 66%, respectively.

The maximum duration of edema seen in this study was five months. No flare‐ups were found in which edema was absent while HO was being formed. In one case (Fig. 1 *A* and *D*), [^18^F]PET/CT did not reveal increased uptake of the tracer yet, whereas the MRI obtained 14 days later, did show moderate edema. Later in the course of this lesion, edema progressed to severe and [^18^F]NaF uptake was found.

## Discussion

The aim of this study was to determine the diagnostic value of MRI in FOP by comparing it with the recently validated marker of disease activity: the [^18^F]NaF PET/CT. Results were evaluated using follow‐up CT and clinical data. In this small series of patients, an active flare‐up was always accompanied by moderate to severe edema on MRI. Interestingly, the presence and severity of edema did not always correlate with the ossifying potency of the involved muscle location. In addition, the chronic stage in FOP was not associated with edema on MRI. Finally, mild edema was seen at several sites, but this was not related to [^18^F]NaF PET/CT, HO, or to clinical complaints.

The finding that all flare‐ups were accompanied by edema on MR‐imaging is in line with five case reports.^9^, ^11^, ^13^, ^14^, ^15^ In these case reports, often MRI was used to evaluate soft tissue swelling in, at that time, an undiagnosed child with FOP. As the course of these flare‐ups was not followed, both implications and diagnostic value of MRI remained unclear. The present study shows a sensitivity of 83% of MRI as compared with [^18^F]NaF PET. Therefore, MRI seems to have potential to rule out acute FOP activity. Chronic FOP activity, however, was not detectable by MRI. This was unexpected, as in a mouse model growth of HO was always associated with edema.^16^ This finding emphasizes the difference between the FOP phenotype in humans and the R206H Acvr1 knock‐in mice.

The specificity to detect HO forming FOP activity using MRI was only 29%, as five edematous lesions did not show increased uptake on [^18^F]NaF PET, nor did these lesions lead to HO progression. The implication of these edematous lesions is still unknown. It most likely is a manifestation of FOP itself, as MRI studies in healthy individuals did not or only sporadically showed edematous lesions.^20^, ^21^ One might hypothesize that lesions with mild edema might reflect a very early stage of disease in which inflammation might still resolve spontaneously. In the current data set, only 6 months CT follow‐up data were available. Whether these mild lesions develop into either the acute or the chronic stage beyond these 6 months remains unclear and will need further investigation.

MRI was compared with [^18^F]NaF PET, a modality that was introduced recently as a method to quantify disease activity.^6^, ^7^, ^8^ [^18^F]NaF PET detects metabolic changes within tissues, as the [^18^F]NaF‐ion binds to newly formed hydroxyapatite.^22^ MRI, on the other hand, detects inflammation through the presence of edema.^10^ In the present study, the intensity of edema did not appear to correlate with the uptake of [^18^F]NaF PET, nor with HO formation or progression. One should take into account that prednisolone was used during flare‐ups to suppress inflammation, which may have interfered with the results.

Although [^18^F]NaF PET is an established technique to evaluate ossifying disease activity in FOP, MRI (if validated in FOP) would be especially useful in pediatric patients, as it does not involve exposure to radiation. In addition, MRI is more widely available than PET. Finally, MRI might be informative in the very early detection of a flare‐up as inflammation is the first stage of FOP lesion formation,^23^ as found in one lesion in the current data set.

The results of the present study indicate that both MRI and [^18^F]NaF are able to identify the acute stage in FOP, flare‐ups, but that MRI is not suitable for identifying the chronic stage, progression of HO in the absence of any clinical signs. There is, however, a third stage, characterized by mild edema (Table 3), which requires further investigation as it is not fully understood. This stage might be a reversible stage that either develops into the acute or chronic stage (moderate to severe edema and [^18^F]NaF PET‐positive) or resolves without HO growth.

**Table 3:** Proposed Stages of FOP Activity Based on MRI and [^18^F]NaF PET/CT Findings According to Eekhoff and Botman

Stages	MRI edema	[^18^F]NaF PET/CT activity	FOP disease stage
0	−	−	No FOP activity
1	+	−	Inflammatory stage
2	−	+	Chronic stage
3	+	+	Acute stage

Another finding of the present study was the long duration of edema present at flare‐up sites. Edema was observed for five months after onset of a flare‐up. In previously reported cases, MRI was performed only within two to five weeksafter the onset of a flare‐up.^(11,15)^ Therefore, it seems that the inflammation that coincides with a flare‐up might be present for a longer period. The significance and explanation of this long duration of edema needs to be further investigated.

The MRI rating used in this study (mild, moderate, or severe) is based on previous studies.^17^, ^19^ The degree of muscle inflammation was based on the overall impression of the muscle (eg, swelling) caused by the inflammation.^17^ Both in the present and in previous studies the agreement between two readers was moderate to good.^17^

The present study is limited by its retrospective design, resulting in different protocols used for MRI. The resolution of images differs only slightly between imaging protocols, it is therefore likely that this has had no influence on our judgment of the edema. In addition, in one patient a field strength of 1.5 Tesla and 3 Tesla were both used. In STIR sequences, however, differences in field strength are not expected to affect the appearance of edema.^24^ A further limitation was the availability of longitudinal [^18^F]NaF PET/CT and MRI scans of only four patients. This is because of the extreme rarity of the disease, the poor mobility of patients, and the unpredictability of flare‐ups. One could also argue whether newly formed cartilage could be misdiagnosed for edema, but this is unlikely because compared with edema, cartilage has lower signal intensity, is more compact, and shows structural distortion of muscle morphology.^25^, ^26^

In conclusion, this study is the first to compare longitudinal imaging data between MRI and [^18^F]NaF PET/CT to establish the diagnostic value of MRI in FOP. MRI is particularly helpful in identifying muscle edema in FOP patients, indicating a concomitant inflammatory process. Moderate and severe edema resulted in the formation of HO in six out of seven lesions. However, only half of the edematous lesions on MRI eventually developed into HO. Moreover, MRI could not detect chronic FOP disease. The significance of mild edematous lesions on MRI that were not related to HO or [^18^F] NaF PET/CT needs further exploration.

## Disclosures

All authors state that they have no conflicts of interest.

## References

[1] Cohen RB , Hahn GV , Tabas JA , et al. The natural history of heterotopic ossification in patients who have fibrodysplasia ossificans progressiva. A study of forty‐four patients. J Bone Joint Surg Am. 1993 Feb;75(2):215–9. 8423182 10.2106/00004623-199302000-00008

[2] Kaplan FS , Le Merrer M , Glaser DL , et al. Fibrodysplasia ossificans progressiva. Best Pract Res Clin Rheumatol. 2008 Mar;22(1):191–205. 18328989 10.1016/j.berh.2007.11.007 PMC2424023

[3] Bravenboer N , Micha D , Triffit JT , et al. Clinical utility gene card for: fibrodysplasia ossificans progressiva. Eur J Hum Genet. 2015 Oct;23(10):1431. 10.1038/ejhg.2014.274 PMC4592076 25604857

[4] Rogers JG , Geho WB . Fibrodysplasia ossificans progressiva. A survey of forty‐two cases. J Bone Joint Surg Am. 1979 Sep;61(6A):909–14. 113413

[5] Pignolo RJ , Bedford‐Gay C , Liljesthrom M , et al. The natural history of flare‐ups in Fibrodysplasia Ossificans Progressiva (FOP): a comprehensive global assessment. J Bone Miner Res. 2016 Mar;31(3):650–6. 27025942 10.1002/jbmr.2728 PMC4829946

[6] Eekhoff EMW , Botman E , Coen Netelenbos J , et al. [18F]NaF PET/CT scan as an early marker of heterotopic ossification in fibrodysplasia ossificans progressiva. Bone. 2018 Apr;109:143–6. 28826841 10.1016/j.bone.2017.08.012

[7] Eekhoff EMW , Netelenbos JC , de Graaf P , et al. Flare‐up after maxillofacial surgery in a patient with Fibrodysplasia ossificans progressiva: an [18F]‐NaF PET/CT study and a systematic review. JBMR Plus. 2018;2(1):55–8. 30283890 10.1002/jbm4.10008 PMC6124206

[8] Botman E , Raijmakers P , Yaqub M , et al. Evolution of heterotopic bone in fibrodysplasia ossificans progressiva: an [(18)F]NaF PET/CT study. Bone. 2019 Jul;124:1–6. 30858149 10.1016/j.bone.2019.03.009

[9] Lin FY , Lin CH , Shu G , Chen CK . Fibrodysplasia ossificans progressiva: initial presentation with a preosseous lesion of the scalp and its MRI appearance. Skeletal Radiol. 2016 Jul;45(7):991–6. 27003387 10.1007/s00256-016-2359-x

[10] Al Mukaddam M , Rajapakse CS , Pignolo RJ , Kaplan FS , Smith SE . Imaging assessment of fibrodysplasia ossificans progressiva: qualitative, quantitative and questionable. Bone. 2018 Apr;109:147–52. 28822792 10.1016/j.bone.2017.08.011

[11] Hagiwara H , Aida N , Machida J , Fujita K , Okuzumi S , Nishimura G . Contrast‐enhanced MRI of an early preosseous lesion of fibrodysplasia ossificans progressiva in a 21‐month‐old boy. AJR Am J Roentgenol. 2003 Oct;181(4):1145–7. 14500246 10.2214/ajr.181.4.1811145

[12] Shiva Kumar R , Keerthiraj B , Kesavadas C . Teaching neuroImages: MRI in fibrodysplasia ossificans progressiva. Neurology. 2010 Feb;74(6):e20. 20142609 10.1212/WNL.0b013e3181cef7d1

[13] Caron KH , DiPietro MA , Aisen AM , Heidelberger KP , Phillips WA , Martel W . MR imaging of early fibrodysplasia ossificans progressiva. J Comput Assist Tomogr. 1990 Mar‐Apr;14(2):318–21. 2312870 10.1097/00004728-199003000-00035

[14] Hamilton SW , Roxburgh C , Renshaw PR . Fibrodysplasia ossificans progressiva: a new spotlight on an old disease‐a case report. Acta Orthop. 2008 Jun;79(3):449–51. 18626811 10.1080/17453670710015391

[15] Merchant R , Sainani NI , Lawande MA , Pungavkar SA , Patkar DP , Walawalkar A . Pre‐ and post‐therapy MR imaging in fibrodysplasia ossificans progressiva. Pediatr Radiol. 2006 Oct;36(10):1108–11. 16932921 10.1007/s00247-006-0270-7

[16] Upadhyay J , Xie L , Huang L , et al. The expansion of heterotopic bone in fibrodysplasia ossificans progressiva is activin A‐dependent. J Bone Miner Res. 2017 Dec;32(12):2489–99. 28782882 10.1002/jbmr.3235

[17] Davis WR , Halls JE , Offiah AC , Pilkington C , Owens CM , Rosendahl K . Assessment of active inflammation in juvenile dermatomyositis: a novel magnetic resonance imaging‐based scoring system. Rheumatology (Oxford). 2011 Dec;50(12):2237–44. 21972421 10.1093/rheumatology/ker262

[18] Andersson H , Kirkhus E , Garen T , Walle‐Hansen R , Merckoll E , Molberg Ø . Comparative analyses of muscle MRI and muscular function in anti‐synthetase syndrome patients and matched controls: a cross‐sectional study. Arthritis Res Ther. 2017 Jan;19(1):17. 28122635 10.1186/s13075-017-1219-y PMC5264447

[19] Kubinova K , Mann H , Vencovsky J . MRI scoring methods used in evaluation of muscle involvement in patients with idiopathic inflammatory myopathies. Curr Opin Rheumatol. 2017 Nov;29(6):623–31. 28796007 10.1097/BOR.0000000000000435

[20] Morrow JM , Matthews E , Raja Rayan DL , et al. Muscle MRI reveals distinct abnormalities in genetically proven non‐dystrophic myotonias. Neuromuscul Disord. Aug 2013;23(8):637–46. 23810313 10.1016/j.nmd.2013.05.001 PMC3744809

[21] Kim HK , Serai S , Lindquist D , et al. Quantitative skeletal muscle MRI: part 2, MR spectroscopy and T2 relaxation time mapping‐comparison between boys with Duchenne muscular dystrophy and healthy boys. AJR Am J Roentgenol. 2015 Aug;205(2):W216–23. 26204310 10.2214/AJR.14.13755

[22] Segall G , Delbeke D , Stabin MG , et al. SNM practice guideline for sodium 18F‐fluoride PET/CT bone scans 1.0. J Nucl Med. 2010 Nov;51(11):1813–20. 21051652 10.2967/jnumed.110.082263

[23] Shore EM . Fibrodysplasia ossificans progressiva: a human genetic disorder of extraskeletal bone formation, or—how does one tissue become another? Wiley Interdiscip Rev Dev Biol. 2012 Jan‐Feb;1(1):153–65. 22408652 10.1002/wdev.9 PMC3297114

[24] Sormaala MJ , Ruohola JP , Mattila VM , Koskinen SK , Pihlajamaki HK . Comparison of 1.5T and 3T MRI scanners in evaluation of acute bone stress in the foot. BMC Musculoskelet Disord. 2011;12:128. 21645348 10.1186/1471-2474-12-128 PMC3121660

[25] Totterman S , Weiss SL , Szumowski J , et al. MR fat suppression technique in the evaluation of normal structures of the knee. J Comput Assist Tomogr. 1989 May‐Jun;13(3):473–9. 2723179 10.1097/00004728-198905000-00020

[26] Paunipagar BK , Rasalkar D . Imaging of articular cartilage. Indian J Radiol Imaging. 2014 Jul;24(3):237–48. 25114386 10.4103/0971-3026.137028 PMC4126138

